# Endogenous controls of gene expression in N-methyl-N-nitrosourea-induced T-cell lymphoma in p53-deficient mice

**DOI:** 10.1186/s12885-017-3536-6

**Published:** 2017-08-14

**Authors:** Xi Wu, Susu Liu, Jianjun Lyu, Shuya Zhou, Yanwei Yang, Chenfei Wang, Wenda Gu, Qin Zuo, Baowen Li, Changfa Fan

**Affiliations:** 1 Division of Animal Model Research, Institute for Laboratory Animal Resources, National Institutes for Food and Drug Control, No. 2 Tiantan Xili, Beijing, 100050 China; 2 National Center for Safety Evaluation of Drugs, National Institutes for Food and Drug Control, Beijing Economic-Technological Development Area, A8 Hongda Middle Street, Beijing, 100176 China

**Keywords:** Reference genes, *p53*-deficient mouse, Lymphoma model, Quantitative real-time PCR

## Abstract

**Background:**

Real-time polymerase chain reaction (PCR) has become an increasingly important technique for gene expression profiling because it can provide insights into complex biological and pathological processes and be used to predict disease or treatment outcomes. Although normalized data are necessary for an accurate estimation of mRNA expression levels, several pieces of evidence suggest that the expression of so-called housekeeping genes is not stable. This study aimed to validate reference genes for the normalization of real-time PCR in an N-methyl-N-nitrosourea (MNU)-induced T-cell lymphoma mouse model.

**Methods:**

T-cell lymphomas were generated in p53-deficient mice by treatment with 37.5 mg/kg MNU. Thymus and spleen were identified as the primary target organs with the highest incidences of lymphomas. We analyzed the RNA expression levels of eight potential endogenous reference genes (*Gapdh*, *Rn18s*, *Actb, Hprt*, *B2M*, *Rplp0*, *Gusb*, *Ctbp1*). The expression stabilities of these reference genes were tested at different time points after MNU treatment using geNorm and NormFinder algorithms.

**Results:**

A total of 65% of MNU-treated mice developed T-cell lymphomas, with the spleen and thymus as the major target organs. All candidate reference genes were amplified efficiently by quantitative reverse-transcription polymerase chain reaction (RT-qPCR). Gene stability evaluation after MNU treatment and during lymphomagenesis revealed that *Ctbp1* and *Rplp0* were the most stably expressed genes in the thymus and spleen, respectively. RT-PCR of thymus RNA using two additional sets of primer confirmed that *Ctbp1* was the most stable of all the candidate reference genes.

**Conclusions:**

We provided suitable endogenous controls for gene expression studies in the T-cell lymphoma model.

**Electronic supplementary material:**

The online version of this article (doi:10.1186/s12885-017-3536-6) contains supplementary material, which is available to authorized users.

## Background

RT-qPCR is a powerful tool for quantifying gene expression and for validating results obtained by other techniques, such as microarray or RNA sequencing [1]. However, a suitable normalization method is necessary to detect variations in the expression levels of specific genes. Normalization usually involves selecting one or more so-called housekeeping genes as reference genes, such as *Gapdh*, *Rn18s*, or *Actb* [2–4]. However, some studies have reported extensive variations in the expression levels of putative reference genes among different tissues and stages of development, as well as in response to experimental treatments. For instance, *Gapdh* and *Actb* showed relatively unstable expression patterns in monosodium L-glutamate-induced obese mice [5], while *Rn18s* and *Actb* showed poor stability in colon cancer [6]. The precise evaluation of gene expression levels thus requires the selection of appropriate reference gene(s) for RT-qPCR analysis according to the particular experimental system.

T-cell lymphoma is an aggressive hematologic tumor resulting from the malignant transformation of T-cell progenitors [7]. Patients with T-cell lymphoma tend to present with very high circulating blast cell counts, mediastinal masses, and central nervous system involvement [8]. Despite a gradual increase in 5-year relapse-free survival rates following intensive chemotherapy, further advances in treatment outcomes require a better understanding of the mechanisms responsible for T-cell lymphoma [9]. T-cell lymphoma and B-cell precursor acute lymphoblastic leukemia have distinct clinical and laboratory features. Understanding the specific gene expression patterns may not only provide insights into the complex biological and pathological processes, but also help to predict disease and/or therapeutic treatment outcomes [10–12].

In the present study, we subjected a heterozygous p53-deficient mouse model (B6-Trp53^tm1DAMR^/NIFDC), established on a C57BL/6 background by embryonic stem (ES) cell targeting, to the intraperitoneal administration of N-methyl-N-nitrosourea (MNU). This model represents a valuable tool for the study of T-cell lymphoma. RT-PCR is a common method of monitoring changes in gene expression during tumor development, and a reference gene is needed to normalize the expression levels of other genes. To identify suitable reference genes during T-cell lymphoma development, we investigated the expression stabilities of eight commonly used candidate reference genes (*Gapdh*, *Rn18s*, *Actb*, *Hprt*, *B2M*, *Rplp0*, *Gusb*, and *Ctbp1*) by RT-qPCR at different time points following the administration of MNU.

## Methods

### Generation of *p53* gene knockout mice and genotyping

A mouse *p53* gene-targeting vector was constructed using a PGK promoter to drive the expression of a neomycin selection cassette (Neo). The targeting vector was introduced into C57BL/6 mouse ES cells by electroporation. After homologous recombination, the targeting vector replaced the *p53* gene from exon 2 to 5. Neomycin resistant ES cell colonies were selected, screened by PCR, and injected into 151 wild-type BALB/c blastocysts. ES-cell-injected blastocysts were then transferred to 14 pseudo-pregnant mice and 8 chimeric mice were produced. Tail genomic DNA was isolated using a Tissue Genomic DNA Extraction Kit (Generay, Shanghai, China) and then subjected to PCR to verify deletion of the *p53* gene. Genomic DNA of *p53* deficient mice and wild-type mice were amplified with primer sets 1 (P53-WT-F, AGTTCTGCCACGTGGTTGGT; P53-WT-R, GTCTCCTGGCTCAGAGGGAG) or 2 (P53-WT-F, AGTTCTGCCACGTGGTTGGT; P53-Neo-R, CAGAGGCCACTTGTGTAGCG), with expected PCR products of 281 bp or 441 bp for wild-type and homozygous mutations, respectively. The male chimera mice were crossed with wild-type C57BL/6 female mice to generate heterozygous *p53* gene knockout mice. For the heterozygous mutation, both bands were visible. C57BL/6 and BALB/c mice were produced in our breeding colony in Institute for Laboratory Animal Resources, National Institutes for Food and Drug Control (NIFDC). ES cell line used in this study was established from C57BL/6 mice in our lab. Blastocysts were obtained by standard protocol from BALB/c mice in our lab.

### MNU-induced malignant lymphoma in *p53*^+/−^ mice

Fifty *p53* ^+/−^-deficient mice were divided into two groups and administered 37.5 mg/kg MNU or citrate buffer (control). MNU was dissolved in citrate-buffered saline and adjusted to pH 4.5 [13] before single intraperitoneal administration on day 1. Five mice from each group were sacrificed immediately and at 4, 8, and 12 weeks after the administration. Thymus and spleen, which were the main tumor target organs, were dissected for histopathological examination and RNA extraction.

### Immunohistochemical analysis

Mouse tissues were fixed in 10% neutral buffered formalin, embedded in paraffin, and sectioned to about 5 μm and stained with hematoxylin and eosin (H&E) for histopathological examination.

Formalin-fixed, paraffin-embedded sections of thymus and spleen were processed for immunohistochemistry. Antibodies directed against CD3 (T-lymphocyte marker), CD20 (B-lymphocyte marker), and CD68 (macrophage marker) were used to classify the lineage of neoplastic cells in the thymus. Thymic malignant lymphoma or thymic sections for CD3, CD20, and CD68 staining were pretreated by incubation at 96 °C in Citra buffer (Zhongshan Golden Bridge Biocompany, Beijing, China) at pH 6 in a microwave for 10 min. Sections for CD3 staining were incubated with anti-CD3 antibody (clone LN10; Zhongshan Golden Bridge Biocompany), at 1:150 dilution, overnight at 4 °C after blocking with normal goat serum for 60 min at 37 °C. Sections for CD20 staining were incubated with anti-CD20 antibody (clone EP7; Zhongshan Golden Bridge Biocompany), at 1:200 dilution, overnight at 4 °C after blocking with normal goat serum for 60 min at 37 °C. Sections for CD68 staining were incubated with anti-CD68 antibody (clone PG-M1; Zhongshan Golden Bridge Biocompany), at 1:200 dilution, overnight at 4 °C after blocking with normal goat serum for 60 min at 37 °C. CD3, CD20, and CD68 immunoreactivities were all detected using a biotinylated rabbit anti-rat secondary antibody followed by an avidin-biotin-horseradish peroxidase complex, and visualized with diaminobenzidine. All immunohistochemical sections were counterstained with hematoxylin, dehydrated in graded concentrations of ethanol, and cover-slipped routinely using permanent mounting medium.

### Tissue RNA extraction and cDNA synthesis

Spleen and thymus were dissected from the mice, immersed immediately in RNAlater stabilization reagent (Invitrogen, Carlsbad, CA, USA), and stored at −80 °C. Grinding of the tissues of three independent mice was performed in liquid nitrogen, followed by homogenization in TRIzol (Invitrogen). Total RNA was extracted in accordance with the manufacturer’s instructions. The amount of total RNA was determined by measuring the absorbances at 260 and 280 nm using a NanoDrop Spectrophotometer (Thermo Fisher Scientific, Waltham, MA, USA). All samples had an A_260_/A_280_ absorption ratio > 1.8. The RNA samples were reverse-transcribed to cDNA in a Reverse Transcription reaction mix using random hexamer primers, in accordance with the manufacturer’s instructions (Takara Bio Inc., Kusatsu, Japan).

### Primer design and RT-qPCR

Primers for RT-qPCR assays of *Gapdh*, *Rn18s*, *Actb, B2M, Hprt*, *Rplp0, Gusb*, and *Ctbp1* were designed using Primer Premier 5.0 (Table 1). Real-time PCR was performed using a Roche LightCycler 480 detection system (Roche Diagnostics, Germany). All standards and samples were run in triplicate in 96-well reaction plates. The cycle conditions were as follows: 15 s template denaturation at 95 °C and then 40 cycles of denaturation at 95 °C for 5 s and elongation at 60 °C for 30 s. This was followed by melting curve analysis, and baseline and cycle threshold values (Ct values) were determined automatically for all plates using Roche LightCycler 480 software.

**Table 1:** Gene-specific RT-qPCR assays

Gene name	Gene symbol	GeneBank No.	Function
Glyceraldehyde3-phosphate dehydrogenase	*Gapdh*	NR_003278.3	Glycolysis pathway enzyme
18S Ribosomal RNA	*Rn18s*	NM_001198859.1	Protein Synthesis
Beta Actin	*Actb*	NM_010368.1	Cytoskeletal structural protein
Beta 2 Microglobulin	*B2m*	NM_007475.5	Beta-chain of major histocompatibility complex
Hypoxanthine phosphoribosyl transferase	*Hprt*	NM_013556.2	Metabolic salvage of purines
Ribosomal Protein large P0	*Rplp0*	NM_009735.3	Structural constituent of ribosome
Beta Glucuronidase	*Gusb*	NM_001289726.1	Glycoprotein, degradation of dermatan and keratin sulfates
C-terminal Binding Protein 1	*Ctbp1*	NM_007393.5	Regulate brown adipose tissue differentiation

### Data analysis

The mRNA expression stability of each candidate gene was analyzed using the freely available Microsoft Excel-based software packages geNorm (https://genorm.cmgg.be/) and Norm-Finder (moma.dk/normfinder-software). Raw Ct values were transformed into relative quantities using the formula 2^−∆ct^. The obtained data were further analyzed using geNorm and NormFinder.

## Results

### Generation of T-cell lymphoma mouse model

**Fig. 1:**
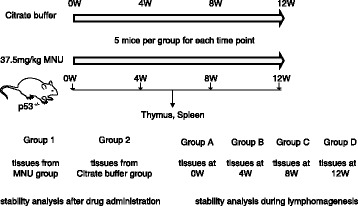
Experimental design of analysis. Fifty *p53* ^+/−^ mice were divided into two groups and administered 37.5 mg/kg MNU or citrate buffer. Five mice from each group were sacrificed immediately and at 4, 8, and 12 weeks after intraperitoneal injection. Thymus and spleen were dissected for RNA extraction. The most stable genes were determined in the MNU and control groups (Groups 1 and 2), and in mice grouped according to the time points after MNU administration (Groups A–D)

**Fig. 2:**
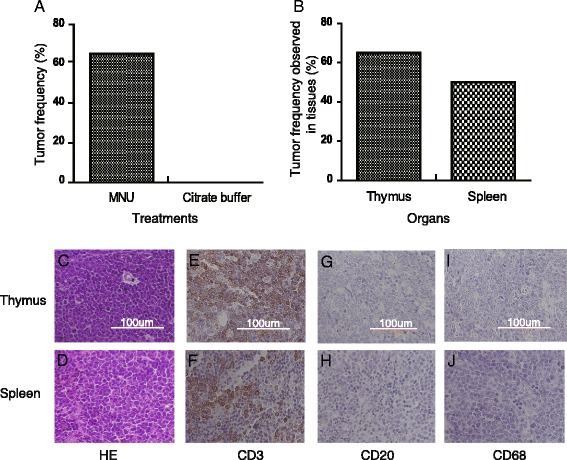
Features of lymphoma occurrence in *p53-*deficient heterozygous mice induced by 37.5 mg/kg MNU. **a** Tumor frequency in mice administered MNU or citrate buffer. **b** Tumor frequencies in thymus and spleen of mice administered MNU. **c** Hematoxylin and eosin (H&E) staining of lymphoma in thymus. **d** H&E staining of lymphoma in spleen. **e**, **f**. Thymus (**e**) and spleen (**f**) lymphoma stained positive for CD3 (T-lymphocyte marker). **g**, **h** Thymus (**g**) and spleen (**h**) lymphoma stained negative for CD20 (B-lymphocyte marker). **i**, **j** Thymus (**i**) and spleen (**j**) lymphoma stained negative for CD68 (macrophage marker). Magnification ×200, scale bar = 100 μm

MNU is a widely used genotoxic carcinogen [13, 14] used to induce T-cell lymphoma in various mouse models, including in the current study (Fig. 1). MNU-treated and control mice were observed twice a week and clinical signs were recorded until sacrifice. Moribund mice were necropsied at the earliest opportunity, and all surviving animals were sacrificed and necropsied at the end of 26 weeks. The thymus and spleen were dissected from the remaining mice for histopathological determination of lymphoma diagnosis and tumor frequency statistics. A total of 65% of MNU-treated mice developed lymphomas, compared with none of the control mice (Fig. 2a). No tumors other than malignant lymphoma were observed. The incidences of lymphomas in the two major target organs were 65% in the thymus and 50% in the spleen (Fig. 2b). Thymus and spleen sections from MNU-treated mice were subjected to hematoxylin and eosin staining (Fig. 2c, d), which showed effacement of thymic corticomedullary architecture by diffuse sheets of lymphoblasts with large euchromatic nuclei, as well as moderate to high numbers of and infiltration of lymphoblasts through the thymic capsule. Besides, immunostaining showed that all neoplastic cells in malignant lymphoma sections were positive for CD3 (Fig. 2e, f) and negative for CD20 (Fig. 2g, h) and CD68 (Fig. 2i, j), indicating that the malignant lymphomas were of T-lymphocyte origin.

### Expression profiles of reference genes

We evaluated the expression stabilities of eight commonly used reference genes (*Gapdh*, *Rn18s*, *Actb, B2M, Hprt1*, *Rplp0, Gusb*, and *Ctbp1*) (Table 1) from different functional classes, to reduce the chance of coregulation of gene expression.

**Fig. 3:**
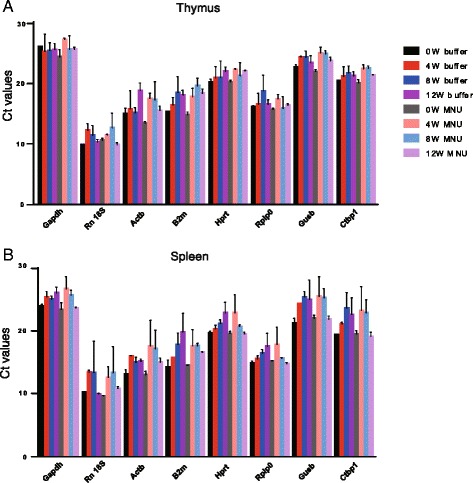
Range of quantification cycle values of the candidate reference genes. Mean of Ct values for the eight reference genes in thymus (**a**) and spleen (**b**) with or without MNU treatment at each time point

The primer sequences and sizes of the amplification fragments are shown in Table 2. Expression stability was assessed by RT-qPCR. The theoretical correlation coefficient (R^2^) for quality assays is 1, but is usually set as >0.980, representing the fit of tested samples to the regression line generated by the standard curve. All primer pairs showed an R^2^ of 0.992–0.999, indicating exponential template duplication. The amplification efficiencies for the eight reference genes ranged from 95 to 105% (Table 2), within the acceptable range of 90%–110% [1], indicating the suitability of the selected primers. Ct values are represented by box-and-whisker plots in Fig. 3. All reference genes displayed similar expression patterns in thymus and spleen, with wide variations in expression levels among different genes. The Rn 18S gene exhibited the lowest mean Ct values in thymus and spleen (10.7 and 11.7, respectively) and *Gapdh* exhibited the highest values (26.2 and 25.0, respectively).

**Table 2:** Selected candidate reference genes

Gene symbol	Primer sequence (5′ → 3′)	Size (bp)	Efficiency (%)	R^2^
*Gapdh*	F1 CAGCAACTCCCACTCTTCCAC R1 TGGTCCAGGGTTTCTTACTC	192	105	0.998
*Rn18s*	F1 GCAATTATTCCCCATGAACG R1 GGCCTCACTAAACCATCCAA	237	95	0.997
*Actb*	F1 CCTCCCTGGAGAAGAGCTATG R1 TTACGGATGTCAACGTCACAC	132	101	0.992
*B2m*	F1 CTCGGTGACCCTGGTCTTTC R1 GGATTTCAATGTGAGGCGGG	170	99	0.998
*Hprt*	F1 AGTCCCAGCGTCGTGATTAG R1 TGATGGCCTCCCATCTCCTT	164	98	0.997
*Rplp0*	F1 CTCTCGCTTTCTGGAGGGTG R1 TCAGTCTCCACAGACAATGCC	172	103	0.994
*Gusb*	F1 TGGGTGTGGTATGAACGGGA R1 GGTCAGTGTGTTGTTGATGGC	123	98	0.999
*Ctbp1*	F1 TGCATGGTACAGTGAGCAGG R1 CTGTAGGCAGCCCCATTGAG	162	105	0.998

### Reference gene stability

**Fig. 4:**
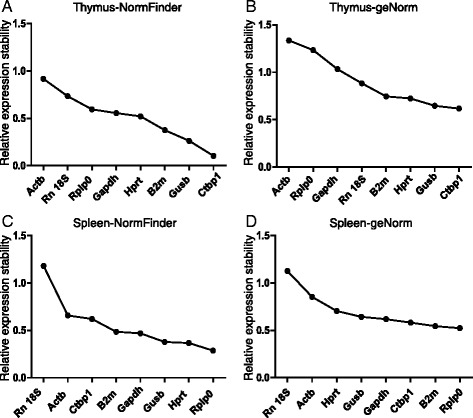
Expression stabilities of the eight candidate genes after administration of MNU. **a**, **b** Mean expression stability values in thymus from least to most stable are presented on the y- and x-axes using geNorm (**a**) and NormFinder (**b**). **c**, **d** Mean expression stability values in spleen from least to most stable expression are presented on the y- and x-axes using geNorm (**c**) and NormFinder (**d**)

We analyzed the data using geNorm and NormFinder to determine the stability of the genes and to identify the most suitable endogenous controls. geNorm and NormFinder are both examples of Microsoft Excel-based software [15]. geNorm analyzes each potential housekeeping gene by comparing its variation with that of all other evaluated reference genes. In contrast, NormFinder separately analyzes sample subgroups and takes into account intra- and intergroup variation for normalization factor calculations. Both algorithms calculate relative expression stability values for each reference gene, and the gene with the lowest stability value is considered the most stable gene. We collected tissues from MNU-treated and control mice at 0, 4, 8, and 12 weeks (Fig. 1) and analyzed expression levels by RT-qPCR at the various time points. Stability values were averaged among the four time points. NormFinder analysis revealed stability values of 0.104–0.918 in the thymus (Fig. 4a). The most stable reference gene in the thymus was *Ctbp1*, followed by *Gusb* and *B2M*, while *Actb* was determined as the least stable gene. geNorm analysis confirmed that *Ctbp1* was the most stable reference gene and *Actb* was the least stable, consistent with the results of NormFinder (Fig. 4b). The stability values in the spleen analyzed by NormFinder were 0.287–1.181 (Fig. 4c). The most stable reference gene in the spleen was *Rplp0*, while Rn 18S was the least stable. The geNorm results were consistent with those of NormFinder (Fig. 4d). Owing to the different algorithms adopted by geNorm and NormFinder, the stability values produced by them could not be compared directly, so the candidate reference genes were ranked according to their stability values evaluated by geNorm and NormFinder (Table 3).

**Table 3:** Ranking of the candidate mRNA reference genes according to their stability value using geNorm and NormFinder

	Thymus				Spleen			
	Time		Drug		Time		Drug	
Gene symbol	NormFinder	geNorm	NormFinder	geNorm	NormFinder	geNorm	NormFinder	geNorm
*Ctbp1*	5	5	6	5	2	4	4	4
*Gusb*	6	8	5	7	8	8	8	8
*Rplp0*	8	7	8	8	4	7	7	7
*Hprt*	7	6	4	3	7	6	2	5
*B2m*	3	3	3	4	3	2	6	2
*Actb*	4	4	7	6	1	1	1	1
*Rn18s*	2	2	2	2	6	3	5	3
*Gapdh*	1	1	1	1	5	5	3	6

**Fig. 5:**
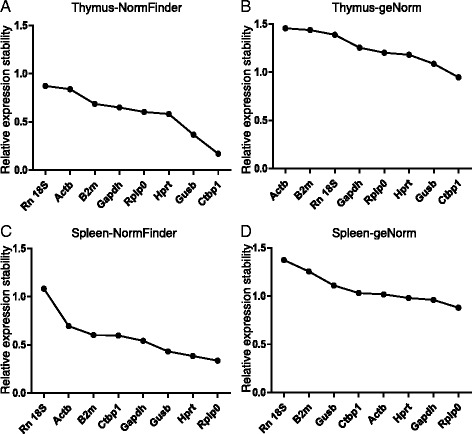
Expression stabilities of the eight candidate genes during lymphoma development. **a**, **b** Mean expression stability values in thymus from least to most stable expression are presented on the y- and x-axes using geNorm (**a**) and NormFinder (**b**). **c**, **d** Mean expression stability values in spleen from least to most stable expression are presented on the y- and x-axes using geNorm (**c**) and NormFinder (**d**)

The stabilities of genes during lymphomagenesis were also determined using NormFinder and geNorm. RT-qPCR data at each time point were grouped together and gene stability was analyzed across the time course. NormFinder analysis revealed stabilities of 0.169–0.873 in the thymus (Fig. 5a). The most stable reference gene was *Ctbp1*, followed by *Gusb* and *Hprt*, while *Rn18s* was the least stable. geNorm also identified *Ctbp1* as the most stable reference gene and *Actb* as the least stable (Fig. 5b). Stability values in the spleen according to NormFinder ranged from 0.336 to 1.083 (Fig. 5c). The most stable reference gene in the spleen was *Rplp0*, while *Rn18s* was the least stable. The geNorm results were consistent with those of NormFinder (Fig. 5d).

To rule out the possibility that the result was dependent on the specific primer used, two additional primers were designed for each reference gene. Sequence and amplification efficiencies are shown in Additional file 1: Table S1. RT-qPCR was then performed for thymus RNA using the additional primers, and mean Ct values of the primers at each time point are presented in Additional file 1: Figure S1. Raw Ct values were transformed into relative quantities using the formula 2^−∆ct^. The obtained data were further analyzed using geNorm and NormFinder. Consistent with our previous result, *Ctbp1* was found to be the most stable gene both after MNU treatment (Additional file 1: Figure S2) and during lymphomagenesis (Additional file 1: Figure S3).

## Discussion

The rapid increase in the incidence of T-cell lymphoma and its poor prognosis highlight the need for a reliable animal model to study the mechanisms by which this disease develops. We thus established a mouse T-cell lymphoma model by MNU induction. The current and previous reports indicate that the thymus and spleen are the target organs with the highest rates of lymphoma in such a model [13]. Monitoring changes in gene expression profiles during T-cell lymphoma development may provide clues to key genetic events, and may help to identify potential biomarkers for the diagnosis of T-cell lymphoma. RT-qPCR has been used widely to detect changes in gene expression because of its high accuracy and convenient methodology. However, it is essential to choose suitable reference genes for normalizing RT-qPCR data to ensure that the results reflect the true relative transcript abundances of genes within cells and tissues [16]. Although endogenous reference genes are widely used, few studies have examined the expression stability of such genes during tumorigeneses.

Previous studies evaluated the selection and effect of controls on normalized gene expression data; however, most of these involved human samples [17, 18]. We analyzed eight commonly used reference genes across T-cell lymphoma target tissues during different stages of tumorigenesis in an MNU-treatment animal model. The results of the current study indicated that the expression levels of so-called housekeeping genes were not stable, but were influenced by the stage of the lymphoma, tissue type, and MNU treatment. geNorm and NormFinder use different strategies to evaluate reference genes, and we therefore used both of these in the current study. Both analyses identified the same reference genes as the most stable after MNU induction and tumorigeneses. *Ctbp1* and *Rplp0* were selected as the best reference genes for the thymus and spleen, respectively, while *Rn18s* was considered to be the least stable gene. Other studies have also reported different stable genes in different tissues [19].

*Ctbp* plays central roles in both development and disease [20]. *Ctbp1* and *Ctbp2* are closely related genes that act as transcriptional corepressors. *Ctbp*s primarily exert transcriptional repression through the recruitment of a corepressor complex to DNA. *Ctbp* overexpression has been observed in many human cancers, resulting in increased epithelial–mesenchymal transition, cancer cell survival, and stem cell-like features [21]. However, *Ctbp1* exhibited a stable expression profile in the current T-cell lymphoma model. Further studies are needed to explore the precise functions of this gene.

Several programs are available for evaluating the stability of candidate genes, including GeNorm, NormFinder, and BestKeeper [15, 22, 23]. We increased the reliability of the results in the present study by using both GeNorm and NormFinder. The orders of stability of the less stable candidate reference genes were not completely consistent between NormFinder and geNorm, which could be explained by the different principles that they use. The model-based approach used by NormFinder has the advantage of being able to differentiate between intragroup and intergroup variation, making it a suitable tool for identifying candidate genes when different sample groups are assessed. However, it has the disadvantage of requiring larger sample sizes than geNorm (>8). In contrast, the pairwise correlation used by the geNorm algorithm is known to be a strong algorithm for small sample sizes.

## Conclusions

In conclusion, we identified *Ctbp1* and *Rplp0* as the best reference genes for thymus and spleen, respectively, in an MNU-induced T-cell lymphoma mouse model. To the best of our knowledge, this study provides the first systemic evaluation of reference genes in a mouse model of lymphoma.

## Additional file

Additional file 1: Stability analysis of reference genes in thymus by two additional primers. To rule out the possibility that the result was dependent on the specific primer used, two additional primers were designed for each reference gene. Sequence and amplification efficiencies, mean Ct values of the primers at each time point and result analyzed using geNorm and NormFinder were presented in the file. **Figure S1.** Range of quantification cycle values of the candidate reference genes. Mean of Ct values of primer 2 (A) and primer 3 (B) for the eight reference genes in thymus with or without MNU treatment at each time point. **Figure S2.** Expression stabilities of the eight candidate genes after MNU treatment in thymus. A, B. Mean expression stability values in thymus from least to most stable are presented on the y- and x-axes using geNorm (A) and NormFinder (B). **Figure S3.** Expression stabilities of the eight candidate genes during lymphoma development in thymus. A, B. Mean expression stability values in thymus from least to most stable expression are presented on the y- and x-axes using geNorm (A) and NormFinder (B). (DOCX 413 kb)

## Abbreviations

Ct values: Cycle threshold values
ES: Embryonic stem
H&E: Hematoxylin and eosin
MNU: N-methyl-N-nitrosourea
NIFDC: National Institutes for Food and Drug Control
PCR: Real-time polymerase chain reaction
RT-qPCR: Quantitative reverse-transcription polymerase chain reaction
