# Supplementary material for: Endogenous controls of gene expression in N-methyl-N-nitrosourea-induced T-cell lymphoma in p53-deficient mice
Source: BMC Cancer. 2017 Aug 14;17:545. doi: 10.1186/s12885-017-3536-6 (PMC5557555; doi:10.1186/s12885-017-3536-6)
Supplement: Additional file 1: — Stability analysis of reference genes in thymus by two additional primers. To rule out the possibility that the result was dependent on the specific primer used, two additional primers were designed for each reference gene. Sequence and amplification efficiencies, mean Ct values of the primers at each time point and result analyzed using geNorm and NormFinder were presented in the file. Figure S1. Range of quantification cycle values of the candidate reference genes. Mean of Ct values of primer 2 (A) and primer 3 (B) for the eight reference genes in thymus with or without MNU treatment at each time point. Figure S2. Expression stabilities of the eight candidate genes after MNU treatment in thymus. A, B. Mean expression stability values in thymus from least to most stable are presented on the y- and x-axes using geNorm (A) and NormFinder (B). Figure S3. Expression stabilities of the eight candidate genes during lymphoma development in thymus. A, B. Mean expression stability values in thymus from least to most stable expression are presented on the y- and x-axes using geNorm (A) and NormFinder (B). (DOCX 413 kb) [file 12885_2017_3536_MOESM1_ESM.docx]

|  | Primer sequence(5’ →3’) | size | Efficiency(%) | R^2^ |
| --- | --- | --- | --- | --- |
| Gapdh primer2 | AGGTCGGTGTGAACGGATTTG | 123 | 100.7 | 0.981 |
|  | TGTAGACCATGTAGTTGAGGTCA |  |  |  |
| Gapdh primer3 | TGGCCTTCCGTGTTCCTAC | 178 | 102.1 | 0.996 |
|  | GAGTTGCTGTTGAAGTCGCA |  |  |  |
| Ctbp1 primer2 | GTGCCCTGATGTACCATACCA | 83 | 111 | 0.992 |
|  | GCCAATTCGGACGATGATTCTA |  |  |  |
| Ctbp1 primer3 | CTGGGGATCTAGGCATCGC | 100 | 106.9 | 0.975 |
|  | GTTCGTCGGTACAGGTTCAGG |  |  |  |
| Gusb primer2 | GGCTGGTGACCTACTGGATTT | 131 | 94.5 | 0.979 |
|  | GGCACTGGGAACCTGAAGT |  |  |  |
| Gusb primer3 | ATGGGATTCATGTGGTGGAAC | 113 | 95.4 | 0.982 |
|  | GTTGATGGCAATCGTGATCCG |  |  |  |
| Rplp0 primer2 | AGATTCGGGATATGCTGTTGGC | 109 | 97.5 | 0.997 |
|  | TCGGGTCCTAGACCAGTGTTC |  |  |  |
| Rplp0 primer3 | TGAGATTCGGGATATGCTGTTGG | 110 | 100.3 | 0.998 |
|  | CGGGTCCTAGACCAGTGTTCT |  |  |  |
| Hprt primer2 | TCAGTCAACGGGGGACATAAA | 142 | 104 | 0.994 |
|  | GGGGCTGTACTGCTTAACCAG |  |  |  |
| Hprt primer3 | AGTCCCAGCGTCGTGATTAG | 88 | 100.1 | 0.993 |
|  | TTTCCAAATCCTCGGCATAATGA |  |  |  |
| B2m primer2 | TTCTGGTGCTTGTCTCACTGA | 104 | 100.2 | 0.984 |
|  | CAGTATGTTCGGCTTCCCATTC |  |  |  |
| B2m primer3 | TTCTGGTGCTTGTCTCACTGA | 104 | 97.6 | 0.964 |
|  | CAGTATGTTCGGCTTCCCATTC |  |  |  |
| Actb primer2 | GGCTGTATTCCCCTCCATCG | 154 | 96.6 | 0.998 |
|  | CCAGTTGGTAACAATGCCATGT |  |  |  |
| Actb primer3 | GTGACGTTGACATCCGTAAAGA | 245 | 103 | 0.992 |
|  | GCCGGACTCATCGTACTCC |  |  |  |
| 18s primer2 | TGGGCCTGCGGCTTAATTTG | 102 | 120% | 0.998 |
|  | CACCACCCACGGAATCGAGA |  |  |  |
| 18s primer3 | CCGGACACGGACAGGATTGA | 128 | 100 | 0.998 |
|  | GCATGCCAGAGTCTCGTTCG |  |  |  |

Table S1 Sequence and amplification efficiency of two primers of candidate reference genes


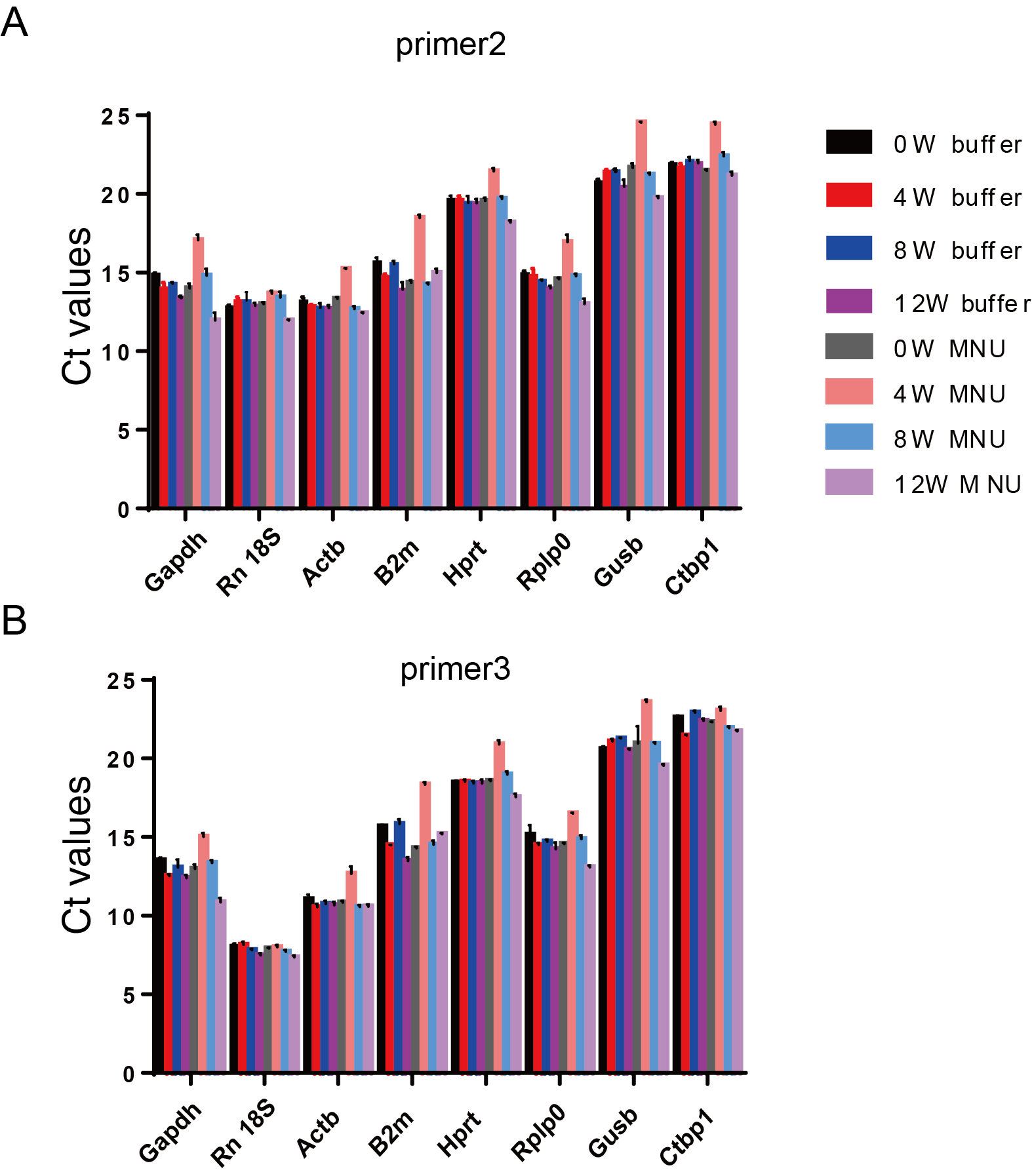


Figure S1 Range of quantification cycle values of the candidate reference genes

Mean of Ct values of primer2 (A) and primer3 (B) for the eight reference genes in thymus with or without MNU treatment at each time point.


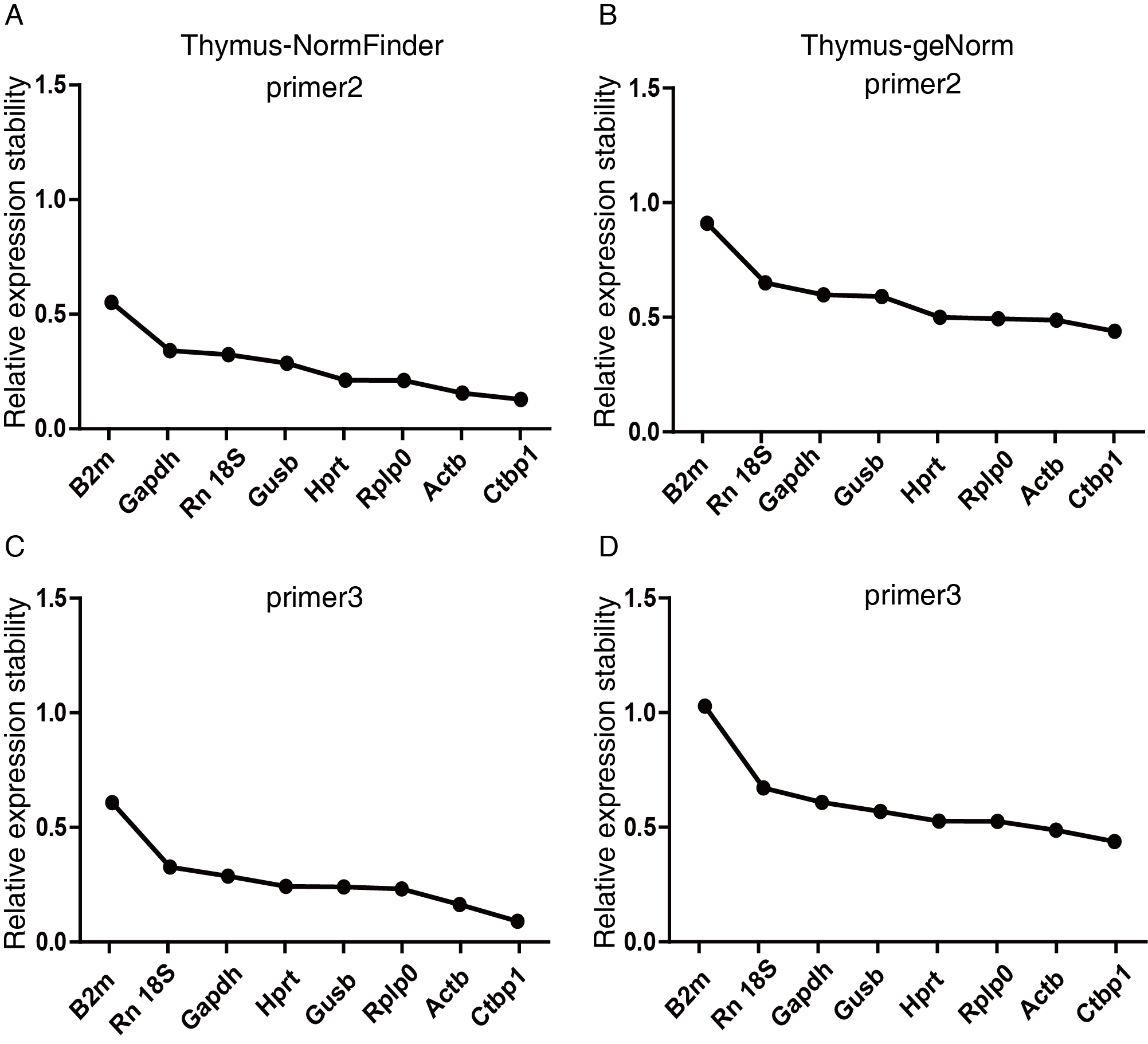


Figure S2. Expression stabilities of the eight candidate genes after MNU treatment in thymus

(A and B) Mean expression stability values in thymus from least to most stable are presented on the y- and x-axes using geNorm (A) and NormFinder (B)


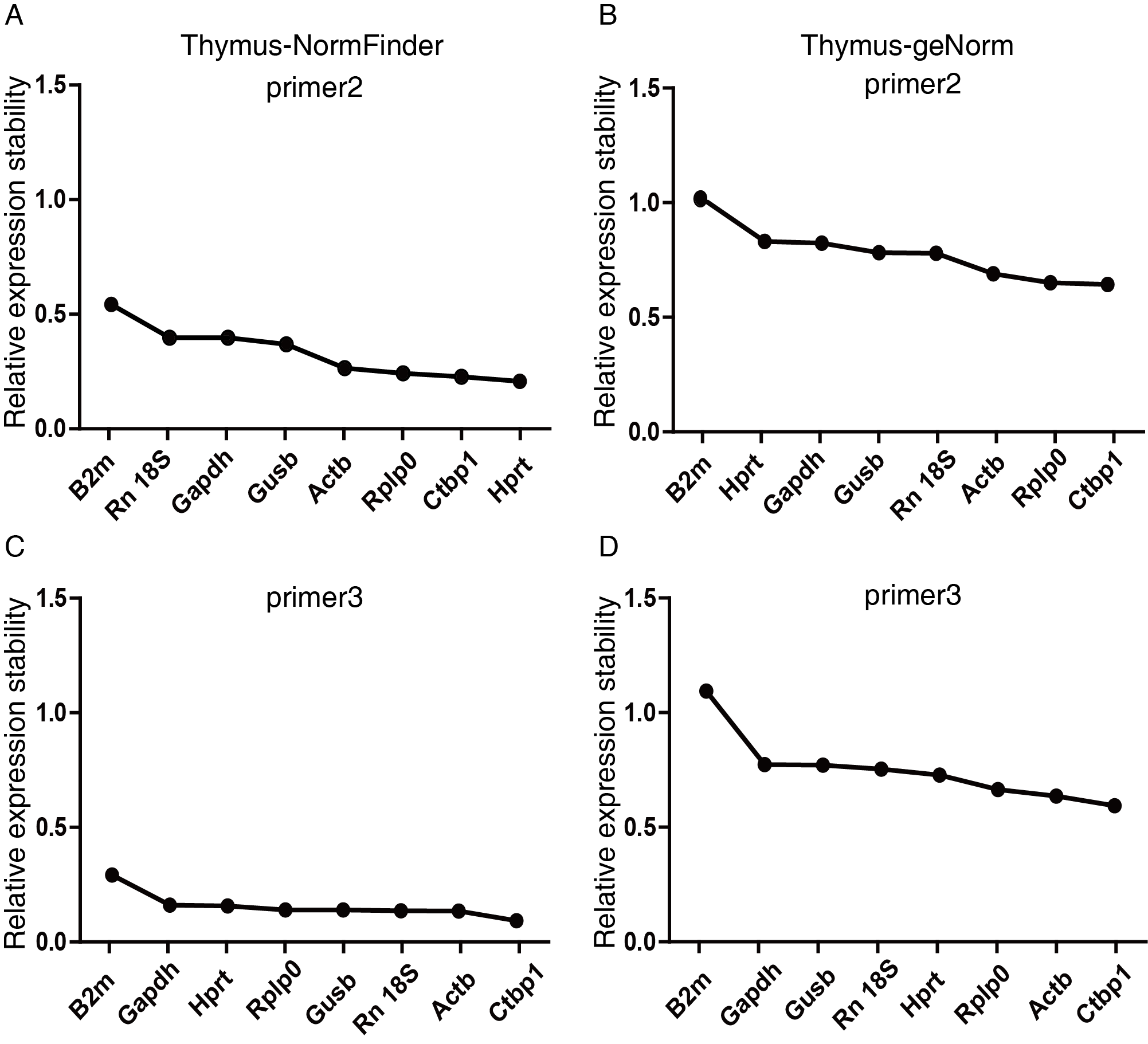


Figure S3. Expression stabilities of the eight candidate genes during lymphoma development in thymus

(A and B). Mean expression stability values in thymus from least to most stable expression are presented on the y- and x-axes using geNorm (A) and NormFinder (B).

Table S2. Ranking of the candidate mRNA RGs according to their stability value using geNorm and NormFinder

|  | thymus primer2 | | | | thymus primer3 | | | |
| --- | --- | --- | --- | --- | --- | --- | --- | --- |
|  | time | | drug | | time | | drug | |
|  | NormFinder | geNorm | NormFinder | geNorm | NormFinder | geNorm | NormFinder | geNorm |
| *Gapdh* | 6 | 6 | 7 | 6 | 7 | 7 | 6 | 6 |
| *Rn18s* | 7 | 4 | 6 | 7 | 3 | 5 | 7 | 7 |
| *Actb* | 4 | 3 | 2 | 2 | 2 | 2 | 2 | 2 |
| *B2m* | 8 | 8 | 8 | 8 | 8 | 8 | 8 | 8 |
| *Hprt* | 1 | 7 | 4 | 4 | 6 | 4 | 5 | 4 |
| *Rplp0* | 3 | 2 | 3 | 3 | 5 | 3 | 3 | 3 |
| *Gusb* | 5 | 5 | 5 | 5 | 4 | 6 | 4 | 5 |
| *Ctbp1* | 2 | 1 | 1 | 1 | 1 | 1 | 1 | 1 |
